# Oral cancer, HPV infection and evidence of sexual transmission

**DOI:** 10.4317/medoral.18419

**Published:** 2013-03-25

**Authors:** Fátima Martín-Hernán, Juan G. Sánchez-Hernández, Jorge Cano, Julián Campo, Jorge del Romero

**Affiliations:** 1 DDS, Universidad Complutense de Madrid. Private practice. Madrid; 2 DDS, PhD, Department of Medicine and Orofacial Surgery. Universidad Complutense de Madrid; 3 MD, MSc, Sandoval Centre for Sexually Transmitted Infections. Madrid

## Abstract

The incidence of oropharyngeal cancer and oral cancer is growing worldwide, both in young non-smokers and in young non-drinkers (smoking and drinking are considered the main risk factors). Epidemiologic studies suggest a strong association between the infection by human papillomavirus (HPV), especially types 16 and 18 (high oncological risk) which have already demonstrated their etiological role in anal tumours as well as in cervix cancer. There is clear epidemiologic evidence that both types of tumours relate to changes in sexual behaviour and that both are linked to sexual transmission of HPV. The number of oral and oropharyngeal cancer cases is rising nowadays, especially among young individuals with no typical toxic habits, such as tobacco and/or alcohol. In this review we set out to update the aspects related to the onset of oral cancer, its relationship with HPV infection and whether this association may be due to the sexual transmission of the virus.

** Key words:**Human papillomavirus, oral sex, head and neck cancer, oral cancer, squamous cell carcinoma, oropharyngeal cancer.

## Introduction

Head and neck carcinomas (HNCs) stem from the mucosa lining the oral cavity, the oropharynx, the hypopharynx, the larynx, the nasopharynx and the sino-nasal tract. The most common histological type is squamous cell carcinoma (SCC) (1,2).

Over 650,000 patients are diagnosed with head and neck squamous cell carcinoma (HNSCC) worldwide every year and over 350,000 die of it (3). In the USA, the incidence of HNSCC in pharynx and oral cavity is 11.9 per 100,000, with men affected in a 3:1 proportion compared to women (4); furthermore, the incidence of oropharyngeal carcinoma has stepped up, specifically in the amygdala and in the base of the tongue, and in individuals aged 40-55 (4).

The similarities in the clinical aspects between the oral and genital injuries associated with HPV led clinicians in the eighties to suggest that HPV might be involved in oral carcinogenesis (3). Some of the factors involved would be HPV’s affinity to epithelial cells, HPV’s oncogenic potential and morphological similarities between genital and oropharyngeal epithelia (2). Up until now HPV’s role in HNSCC has been highly controversial, though, chiefly because the widely varying detection rates of the virus’ DNA (from 1% to 100%, depending on the detection method applied) (5). Other possible reasons for confusion may have been regarding HNSCC as a single entity, or studies failing to specify whether the localization was just oral, oropharyngeal or others, which would render it difficult to extrapolate the obtained data (2,3).

Recently, data from case-control and meta-analytic studies show that HPV would be, indeed, an independent risk factor for the development of oropharyngeal and oral carcinomas (3,6-8). HNSCCs associated with HPV seem to be, thus, a distinct clinical entity with a different evolution too, since these tumours have a better prognosis than the HPV-negative ones (1,8).

Moreover, a strong association seems to exist between patient age and HPV-16’s infection prevalence (9). These cases occur mostly in white and Asian males (2) with no previous record of tobacco or alcohol consumption, two risk factors traditionally associated with this infection (1,3).

The association between these oncogenic HPV types (16 and 18) in cervix cancer as well as with anal cancer has been clearly demonstrated, just like the role of certain sexual conducts in their transmission (10). In this sense, evidence is starting to exist suggesting that certain sexual practices, such as oral sex (oral-genital contact) and others (oral-anal contact), and certain sexual conducts (such as having many sexual partners) would favour the virus to reach the oral cavity and be in a position to play a role in the development of neoplasia in the oropharyngeal region (11).

## Material and Methods

In order to carry out this review, a search has been conducted for articles from 2000 up to 2010 related to HNSCC, especially at orpharyngeal and OSCC levels, using keywords such as *“head and neck squamous cell cancer”*; *“HPV” or “oral cancer”*. Articles related to HPV and larynx cancer have been excluded, as they exceed this field. For our purposes, data bases such as *Medline, Pubmed, Current Contents Connect, Cochrane plus and Catálogo Cisne* have been used.

## Results

-Relationship among HPV infection, oral cancer and oropharyngeal cancer. The role of sexual conducts.

Tobacco, alcohol, poor oral hygiene and genetics remain important risk factors in head and neck tumour’s global figures (for individuals aged over 60), although HPV is currently acknowledged as one of the primary causes behind the rise in oropharyngeal cancer cases. In the USA, between 40% and 80% of oropharyngeal cancers are caused by HPV, while in Europe figures vary from Sweden’s 90% to less than 20% found in other communities with a higher prevalence of tobacco consumption (1).

HNC’s total incidence in the USA has diminished over the last years due to a lower consumption of tobacco. Yet the incidence of HPV related oropharyngeal cancer seems to be rising, as shown by SEER (Surveillance Epidemiology and End Results), not only in the USA but also in Sweden, highlighting the relevance of this causal association (1). SEER’s data show a statistically significant growth of oropharyngeal cancer affecting young adults aged between 20 and 44 (12).

As for OSCC, Miller et al. (7) performed a study evaluating the likelihood of finding high oncogenic risk HPV types in patients with OSCC injuries. They found a higher detection prevalence of HPV’s viral DNA in cell samples coming from neoplasias, verrucous carcinomas and OSCC than in healthy mucosa. The probability of finding some high oncological risk types (HPV 16 and 18) was 2.8 times higher, suggesting that these viral types are a significant risk factor in the development of OSCC injuries (7).

Termine et al. (2), in a meta-analytical review of HNSCC cases (oral and others) and of diverse HPV detection techniques concluded that variations in viral DNA prevalence may be affected by the failure to distinguish the tumour’s localization as well as by the detection technique employed (PCR or in-situ hybridization) (2).

Even though it has been known for a long time that HPV is a key etiological factor in anogenital cancer, it is only recently that its etiological relationship with an HNSCC subtype has been proved. There are over 100 different types of HPVs, and at least 15 of these have oncogenic potential. Notwithstanding, most HNSCCs (>90%) are caused by the HPV-16 type, the same type involved in anogenital cancers associated with HPV (1).

On the other hand, HPV prevalence seems lower in OSCC injuries than in oropharyngeal carcinomas (6). The HPV prevalence difference between OSCC cases and oropharyngeal carcinoma in consumers and non-consumers of tobacco and/or alcohol would support the theory stating that these tumours might constitute an etiologically different subgroup within head and neck tumours (13-16).

HPV genital infection is the most common sexually transmitted infection (STI) these days, with an incidence of about 5.5 million worldwide. About 75% of sexually active men and women have been exposed to HPV at some point in their lives (17). HPV types 16 and 18 are responsible for about 70% of cervix, vagina and anal cancers and for about 30% to 40% of vulva, penis and oropharyngeal cancer cases (10,18).

The likely ethiological role of HPV in cancer has been put forward after finding out that the sexual partner of patients diagnosed with an HPV infection presented a higher risk of developing a head and neck cancer (19). However, between 10% and 30% of HPV-positive HNSCCs occur among heavy smokers and/or drinkers, which makes it harder to clearly establish the true etiological role that HPV may play (8).

As has already been said, HPV infection is a STI and the consulted studies suggest that the number of sexual partners in the lifetime would be an important risk factor, not only for anal and cervix cancer, but also for the development of HNSCC (19). In case-control studies, the risk of developing HPV induced HNSCC doubled in individuals who reported having had between one and five sexual partners and increased five times in those who reported having had six or more, compared to those who did not practice oral sex (8,20).

A recent study suggests a rise in HPV infection incidence in young adults, mainly due to an earlier initiation of sex and to a higher number of sex partners (19). In the USA, most school age youngsters report having had oral sex, over two thirds of them with more than one partner, which would be facilitating the exposure of HPV to the oropharyngeal epithelium (9).

Yet it is also important to add that HPV-positive HNSCC cases were present in individuals who had had less sexual partners. Thus, over 50% of patients with HPV-positive tumours reported having had five or less sexual partners while between 8% and 40% reported having never had oral sex. Therefore, even though sexual conducts seem to be a key risk factor for HPV-positive HNSCC, the absence of a high number of sexual partners does not prevent the development of an HPV-positive HNSCC (1).

Both men and women can be asymptomatic HPV carriers and the virus transmission to the oral cavity would mainly happen through unprotected sex practices. Research carried out by D’Souza et al. (19) shows an HPV increase in couples who practiced oral sex (19,20). On the other hand, individuals who have previously had HPV infections are thirty two times more likely to develop malignant injuries than those who have not (20,21).

A thorough study published in 2010 about sexual behaviour and HNC risk carried out by INHANCE (International Head and Neck Cancer Epidemiology Consortium), shows that sexual behaviour is linked to the risk of developing an HPV associated cancer, depending on its localization. Thus, the study found out that oropharyngeal cancer was significantly associated with having had six or more sex partners and four or more oral sex partners (22).

As for amygdala cancer, it was associated with having had four or more oral sex partners; and among men, to having ever had oral sex and to a younger age at the time of their first sexual relationship. As far as tongue base cancer is concerned, it was linked, among women to having had oral sex at least with two partners; among men, to having had sex with other men. Little evidence was found, though, of association between the analysed sexual behaviours and their relationship with larynx and oral cavity cancer (22).

D’Souza et al. (19) found that a history of numerous sexual partners (twenty six or more) was significantly associated with the presence of oropharyngeal cancer. The same happened when oral sex had been practiced with more than six individuals (18-20). This link was even stronger among individuals with HPV-16 oral infection, regardless their tobacco or alcohol consumption (19). HPV oral infection was more strongly related to the number of recent couples who had practiced oral sex than to couples who reported having only had vaginal sex (20,23).

It has also been proved that the prevalence of oral infection caused by high oncological risk HPV is higher in seropositive individuals and that it increases with age, male gender and infection by VHS-2 (24).

HIV-positive individuals also have a higher likelihood of developing an oral infection caused by HPV, of being infected by more than one type of HPV and of HPV being of high oncogenic risk (18). The risk of infection by high oncogenic risk HPV among HIV-positive individuals was thirteen times higher in those who practiced oral sex with more than one person during the previous year (22,24). This might indicate that HPV co-infection with other pathogens would increase the infectivity and transmissibility of both (24).

Lastly, some authors disagree and consider the possibility of HPV infection in HSNCC patients being just a temporal non-causative infection. Acha-Sagredo et al. (25) did not find HPV’s DNA in forty seven patients already treated of OSCC (25).

In this sense, Herrero et al. (10) suggested too that HPV infection is a temporal one, furthermore related to the presence of a malignant or pre-malignant injury and that it should be born in mind that the detection of HPV’s DNA in tumours should not necessarily have to indicate, per se, a causal association (10,16). Despite these data, studies confirming the high frequency of HPV’s DNA detection in HNSCC are far more numerous (5).

-Pathogenic mechanism of oral/oropharyngeal cancer onset in relation with HPV

Human Papillomavirus (HPV) belongs to the *Papillomaviridae* family. The most important ones (of high oncogenic risk) are types 16 and 18 (8,26). Infections by to the high risk types manifest a silent clinic, cause infections and, in a lower proportion, lead to high grade squamous intra-epithelial injuries and cancer (10,26). The cycle starts when viral DNA builds into keratinocytes’ cell genome (2). For a malignant transformation caused by VPH to occur, viral DNA has to penetrate the epithelial cells of the guest in first place. Then the synthesis of E6 and E7 viral proteins takes place due to the disruption of E2 (viral protein). E6 viral protein inactivates p53, on the one hand, and E7 oncoprotein would inactivate the pRb (both are tumour suppressor proteins), producing an increment of the mitotic activity of the affected cell (5,16). All that would lead to genomic instability, to an absence of DNA reparation, to defects in apoptosis and in the regulation of the cell cycle. HPV might contribute both to oncogenesis and to tumour progression. As a result, it would not only be required to suffer from a HPV infection, but both certain mutations in the suppressor genes and the activation of oncogenes caused by the virus must occur, or otherwise have a special genetic susceptibility, for a neoplastic epithelial injury to develop (3,13,16,27).

When an HPV infection happens, antibodies such as HPV-16 L1 as well as HPV-16 E6 and E7 viral proteins show up in serum. These viral proteins are capable of producing a malignant transformation of upper respiratory, anal and genital tracts cells. The risk of developing OSCC when having antibodies against these proteins would be between 1.5 and 3.4 times higher, for the oral cavity, and between 3.5 and 19 times higher, for the oropharynx, than otherwise (10,26).

P16 is another protein involved in cell cycle used by many authors as a likely marker for oral cancer associated with an HPV infection. It has been found out that an over-expression of P16 would be linked to HPV-positive oropharyngeal carcinomas, poorly differentiated and in locally more advanced stages (T4, N2-3) (16). Hence, in HPV-positive malignant injuries the augmented expression of this protein could be used as a favourable prognosis marker (16,28). On the other hand, the loss of the P16 expression by deletion, hypermethylation or mutation is common in OSCCs caused by alcohol and tobacco, producing injuries with a worse prognosis as they do not respond to chemotherapy or radiotherapy in the same way. E6 HPV’s oncoprotein can inactivate P53 too, although in these cases it would be a functional inactivation, not a mutation as it happens in OSCCs associated with tobacco and alcohol intake. In fact, the rate of P53 mutations due to HPV is very low (4,16,28,29).

All this would support the existing evidence of oral/oropharyngeal cancer etiologically associated with HPV having an increased survival and a better prognostic, as both an over-expression of P16 and very low P53 mutation rates would happen, and all this might be linked to infected cells being more sensitive to current radiotherapy and chemotherapy (28,29).  Table 1 shows the differences between HPV-positive and HPV-negative oral cancer.

**Table 1:**
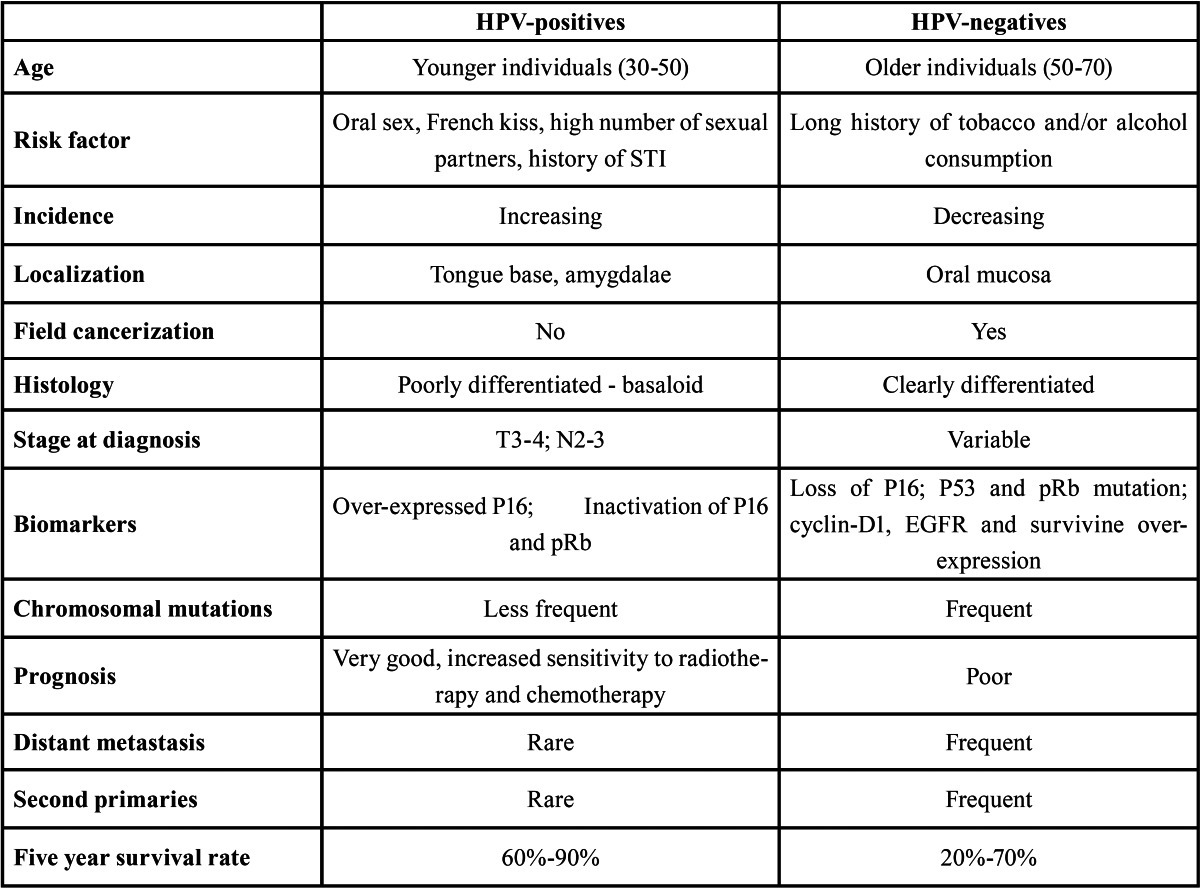
Differences between oropharyngeal cancer HPV + and HPV -.

-Treatment of oral/oropharyngeal cancer associated with HPV. The odontologist’s role in its early prevention and detection.

The average time span from the moment in which HPV infection occurs to the onset of cancer has been estimated as around twelve years, so according to some authors the number of young adults who will develop oropharyngeal cancer is expected to increase over the coming years (30,31).

In line with what has been expressed in the previous section, several authors agree in pointing out that patients who display small size tumours, over-expression of P16 and high HPV viral charge in the tumour sample, have an increased survival in spite of the presence of lymphatic nodules (9,16,28).

Nguyen et al. (9) suggest that patients who satisfy these requirements could be included in some study protocol in order to be treated with radiotherapy only, aiming at reducing the treatment’s morbidity as well as increasing their quality of life.

This modality should be considered experimental, since surgery plus post-operative chemotherapy or radiotherapy remains the treatment of choice in patients with locally advanced HNC (9).

HPV vaccine is the first explicitly designed to prevent virus induced cancer (23,31). HPVs 16 and 18 are the main targets of the currently approved vaccines and the available data confirm the success in the incidence reduction of pre-cancerous cervical injuries for these types (31-33). The vaccine’s efficacy is limited by two factors since not all cancers are caused by HPVs 16 and 18; and there seems to be a requirement of vaccinating young women before they get infected by these two types (31). To be effective, such vaccination should start before “sexual puberty” as adolescents regard oral sex as a safe option to prevent non wanted pregnancies and other STIs (including HIV) (33).

There are two commercially available prophylactic vaccines against HPV today: the bivalent (VPHs 16 and 18) Cervarix® (GSK) and the tetravalent (VPHs 6,11,16 and 18) Gardasil® (Merck). Theoretically, there is no reason for these vaccines to fail to work against these same viruses in different localizations (such as oral cavity, pharynx, larynx or the anogenital region). Proving that the vaccine also prevents oropharyngeal cancer would mean not only a landmark in the prevention of these diseases, but it would also provide the missing link in the chain of evidences with the ultimate proof of HPV induced viral etiology of these tumours (3,33).

Vaccination is approved in females aged 9 to 26. The vaccination primary target population should be females aged 11 and 12. However the vaccine can also be administered to females up to 9 years old and to those aged between 13 and 26 who have been sexually active. There are no available data about the vaccine’s efficacy in individuals aged over 26 (15,17,34).

Clinicians, including professionals involved in oral health should pay attention to the risk of oropharyngeal cancer in young individuals, who neither smoke nor drink, to avoid an unnecessary diagnostic and therapeutic delay. There seems justified, considering all that has been said so far in this paper, the inclusion of questions over sexual practices in the anamnesis of young patients presenting enlarged lymphatic nodules and/or masses in amygdalae and/or sore throat, to rule out a possible underlying HPV associated neoplasia. According to some authors, an early referral to the specialist would reduce morbidity as well as the cost of the treatment (9). Routine exploration of mucous, tongue, and neck palpation ought to be mandatory practices in any oral exploration in order to perform the detection of any potentially malignant injury (35).

So far, recommendations on early detection of oral cancer focused on patient groups who traditionally had a higher risk of developing it, i.e. individuals aged over 60, tobacco and/or alcohol consumers as well as individuals with certain hereditary diseases, such as Fanconi’s anaemia, or with a past history of oral cancer (35).

Brondani, in his editorial published in an Odontology reference review, also wonders what our patients know about HPV, about oral sex and about oral cancer. Even more, he acknowledges that dentists have not been properly educated in these aspects, so he encourages a more detailed study of these subjects, their registration in the patient’s clinic history and to supply information about them to patients (21).

Scientific evidence in this point seems to indicate that we must be educated, and educate our children, on other potential risk factors in the onset of oral cancer, such as non-protected oral sex, and not only about tobacco and/or alcohol consumption, especially to early detect this injuries in adolescents and young adults or, better yet, to try to prevent them (21).

## Conclusion

Patients with HPV associated HNSCC are younger and present reduced tobacco and/or alcohol intakes. There are certain molecular profiles of HPV-positive HNSCCs that differentiate them from HPV-negative cancers, as a result they could be considered a biological entity separated from the rest of HSNCC, and of likely sexual transmission. According to the current literature revised, risk factors associated with this HNSCC variant are similar to those of cervix cancer, including number of sexual partners, younger age at their first sexual intercourse, oral sex practices, a history of genital warts and younger age. Dentists, as health professionals, ought to get more heavily involved in the detection of these factors implied in the etiology and pathogenesis of these injuries in order to contribute to the early detection and prevention of this kind of injuries, especially in young patients with no risky habits traditionally linked to oral cancer, such as tobacco and/or alcohol consumption.

## References

[1] Marur S, D'Souza G, Westra WH, Forastiere AA (2010). HPV- associated heck and neck cancer: a virus- related cancer epidemic. Lancet Oncol.

[2] Termine N, Panzarella V, Falaschini S, Russo A, Matranga D, Lo Muzio L et al (2008). HPV in oral squamous cell carcinoma vs head and neck squamous cell carcinoma biopsies: a meta-analysis (1988â2007). Ann Oncol.

[3] SyrjÃnen S (2010). The role of human papillomavirus infection in head and neck cancers. Ann Oncol.

[4] Ritchie JM, Smith EM, Summersgill KF, Hoffman HT, Wang D, Klussmann JP (2003). Human Papillomavirus infection as a prognostic factor in carcinomas of the oral cavity and oropharynx. Int J Cancer.

[5] Gillison ML, Koch WM, Capone RB, Spafford M, Westra WH, Wu L (2000). Evidence for a causal asociation between human papillomavirus and a subset of head and neck cancers. J Natl Cancer Inst.

[6] Dayyani F, Etzel CJ, Liu M, Ho CH, Lippman SM, Tsao AS (2010). Meta- Analysis of the impact of Human Papillomavirus (HPV) on cancer risk and overall survival in head and neck squamous cell carcinomas (HNSCC). Head & Neck Oncology.

[7] Miller CS, Johnstone BM (2001). Human papillomavirus as a risk factor for oral squamous cell carcinoma: a meta-analysis, 1982-1997. OralSurg Oral Med Oral Pathol Oral Radiol Endod.

[8] Gillison ML, DÂSouza G, Westra W, Sugar E, Xiao W, Begum A (2008). Distinct risk factor profiles for human papillomavirus type-16 positive and human papilomavirus type-16 negative head and neck cancers. J Natl Cancer Inst.

[9] Nguyen NP, Chi A, Nguyen LM, Ly BH, Karlsson U, Vinh â Hung V (2010). Human papillomavirus-associated oropharyngeal cancer: a new clinical entity. QJM.

[10] Herrero R, CastellsaguÃ X, Pawlita M, Lissowska J, Kee F, Balaram P (2003). Human papillomavirus and oral cancer: the internacional agency for research on cancer multicenter study. J Natl Cancer Inst.

[11] D'Souza G, Zhang HH, D'Souza WD, Meyer RR, Gillison ML (2010). Moderate predictive value of demographic and behavioral characteristics for a diagnosis of HPV16-positive and HPV16-negative head and neck cancer. Oral Oncol.

[12] Shiboski CH, Schmidt BL, Jordan RC (2005). Tongue and tonsil carcinoma. Increasing trends in the U.S population ages 20-44 years. Cancer.

[13] Tachezy R, Klozar J, SalÃkovÃ M, Smith E, Turek L, Betka J (2005). HPV and other risk factors of oral cavity/oropharyngeal cancer in the Czech Republic. Oral Dis.

[14] Chocolatewala NM, Chaturvedi P (2009). Role of human papiloma virus in the oral carcinogenesis: An Indian perspective. J Cancer Res Ther.

[15] Smith EM, Swarnavel S, Ritchie JM, Wang D, Haugen TH, Turek LP (2007). Prevalence of human papillomavirus in the oral cavity/oropharynx in a large population of children and adolescents. Pediatr Infect Dis J.

[16] Weinberger PM, Yu Z, Haffty BG, Kowalski D, Harigopal M, Brandsma J (2006). Molecular classification identifies a subset of human papillomavirus- associated oropharyngeal cancers with favorable prognosis. J Clin Oncol.

[17] LjubojeviÄ S (2006). The human papillomavirus vaccines. Acta Dermatovenerol Croat.

[18] Kreimer AR, Alberg AJ, Daniel R, Gravitt PE, Viscidi R, Garrett ES (2004). Oral human papillomavirus infection in adults is associated with sexual behavior and HIV serostatus. J Infect Dis.

[19] D'Souza G, Agrawal Y, Halpern J, Bodison S, Gillison ML (2009). Oral sexual behaviors associated with prevalent oral human papillomavirus infection. J Infect Dis.

[20] D'Souza G, Kreimer AR, Viscidi R, Pawlita M, Fakhry C, Koch WM (2007). Case-control study of human papillomavirus and oropharyngeal cancer. N Engl J Med.

[21] Brondani M (2008). HPV, oral sex, and the risk of oral cancer: food for thought. Spec Care Dentist.

[22] Heck JE, Berthiller J, Vaccarella S, Winn DM, Smith EM, Shan'gina O (2010). Sexual behaviours and the risk of head and neck cancers: a pooled analysis in the International Head and Neck Cancer Epidemiology (INHANCE) consortium. Int J Epidemiol.

[23] Forster A, Wardle J, Stephenson J, Waller J (2010). Passport to promiscuity or lifesaver: press coverage of HPV vaccination and risky sexual behavior. J Health Commun.

[24] Sikora AG, Morris LG, Sturgis EM (2009). Bidirectional association of anogenital and oral cavity/pharyngeal carcinomas in men. Arch Otolaryngol Head Neck Surg.

[25] Acha-Sagredo A, Ruesga MT, Aguirregaviria JI, Aguirre Urizar JM (2010). InfecciÃn por VPH y carcinogÃnesis oral. Med Oral Patol Oral Cir Bucal.

[26] Bosch FX, Lorincz A, Mu-oz N, Meijer CJ, Shah KV (2002). The causal relation between human papillomavirus and cervical cancer. J Clin Pathol.

[27] Licitra L, Perrone F, Bossi P, Suardi S, Mariani L, Artusi R (2006). High â risk papillomavirus affects prognosis in patients with surgically treated oropharyngeal squamous cell carcinoma. J Clin Oncol.

[28] Kumar B, Cordell KG, Lee JS, Worden FP, Prince ME, Tran HH (2008). EFGR, p16, HPV Titer, Bcl- xl and p53, sex and smoking as indicators of response to therapy and survival in oropharyngeal cancer. J Clin Oncol.

[29] Fakhry C, Westra WH, Li S, Cmelak A, Ridge JA, Pinto H (2008). Improved survival of patients with human papillomavirus- positive head and neck squamous cell carcinoma in a prospective clinical trial. J Natl Cancer Inst.

[30] Dahlstrom KR, Li G, Tortolero-Luna G, Wei Q, Sturgis EM (2011). Differences in history of sexual behavior between patients with oropharyngeal squamous cell carcinoma and patients with squamous cell carcinoma at other head and neck sites. Head Neck.

[31] Baden LR, Curfman GD, Morrissey S, Drazen JM (2007). Human Papillomavirus Vaccine- Opportunity and Challenge. N Engl J Med.

[32] Medeiros LR, Rosa DD, da Rosa MI, Bozzetti MC, Zanini RR (2009). Efficacy of human papillomavirus vaccines: a systematic quantitative review. Int J Gynecol Cancer.

[33] Schiller JT, CastellsaguÃ X, Villa LL, Hildesheim A (2008). An update of prophylactic human papillomavirus L1 virus-like particle vaccine clinical trial results. Vaccine.

[34] Lugarini J, Maddalo F (2009). Results of a vaccination campaign against human papillomavirus in the province of La Spezia, Liguria, Italy, March-December 2008. Euro Surveill.

[35] Rethman MP, Carpenter W, Cohen EE, Epstein J, Evans CA, Flaitz CM et al (2010). Evidence-based clinical recommendations regarding screening for oral squamous cell carcinomas. J Am Dent Assoc.

